# Two different types of giant bleb formation following Ahmed Glaucoma valve implantation

**DOI:** 10.1016/j.ajoc.2024.102008

**Published:** 2024-02-09

**Authors:** Ricardo Ugarte, Kazunobu Sugihara, Ichiya Sano, Kana Murakami, Mizuki Iida, Ayaka Shimada, Sho Ichioka, Akiko Harano, Masaki Tanito

**Affiliations:** a Department of Ophthalmology, Shimane University Faculty of Medicine, Izumo, Japan; b GlaucomaSalud: Ophthalological Clinic, Lima, Peru; c Department of Ophthalmology, Guillermo Almenara National Hospital, Lima, Peru

**Keywords:** Posterior enlarged giant bleb, Anterior enlarged giant bleb, Ahmed glaucoma valve, Neovascular glaucoma, Childhood glaucoma, Hess chart, Magnetic resonance imaging, Surgical complication

## Abstract

**Purpose:**

This study aims to present two different types of giant bleb formation following Ahmed Glaucoma Valve (AGV) implantation: an anterior enlarged giant bleb and a posterior enlarged giant bleb.

**Observations:**

In Case 1, a 70-year-old Japanese male underwent AGV implantation for neovascular glaucoma in his right eye (OD). Preoperatively, the patient's intraocular pressure (IOP) and best corrected visual acuity (BCVA) were 23 mmHg and 0.6, respectively, OD, while using 3 antiglaucoma topical medications. Two months post-surgery, the patient began experiencing double vision. Slit lamp evaluation revealed no abnormalities, IOP and BCVA were 24.0 mmHg and 0.8, respectively, OD. A posteriorly enlarged bleb in the superotemporal quadrant OD was found to be causing displacement on T2-weighted orbital MRI. The patient underwent surgical excision of the anterior bleb wall. By three weeks post-surgery, the double vision resolved; IOP and BCVA were 17 mmHg and 0.7, respectively, and a normal bleb in the slit lamp evaluation was identified OD. In Case 2, a 10-year-old Japanese female underwent AGV implantation for childhood glaucoma associated with congenital cataract OD. Preoperatively, IOP and BCVA were 30 mmHg and 0.5, respectively, OD, while using 3 antiglaucoma topical medications. She underwent pars plana vitrectomy (PPV) in addition to AGV implantation. Seven months post-surgery, slip lamp evaluation revealed an anteriorly enlarged giant bleb that only cause her a cosmetic concern.

**Conclusions and Importance:**

There are two types of giant bleb formation following AGV implantation based on the direction of the enlargement: an anterior enlarged giant bleb and a posterior enlarged giant bleb. The introduction of this classification contribute to better understanding and management of this unusual surgical complication.

## 1 Introduction

Although rare, the formation of a giant bleb can occur following glaucoma tube implantation surgery.^1^, ^2^, ^3^, ^4^ Such giant bleb may be associated with symptoms such as pain/foreign body sensation, progressive proptosis, limitation of eye movement, exposure keratitis, dellen formation, and cosmetic problem that may vary among each patient.^1^, ^2^, ^3^, ^4^ Treatment approaches for these conditions also vary, ranging from conservative measures^1^^,^^4^ to surgical intervention.^3^ In this report, we present 2 cases of giant bleb formation following AGV implantation. Based on symptoms, exam findings, auxiliary testing from this case as well as other case reports, blebs morphology can be classified in two different types.

## 2 Case report

### 2.1 Case 1

**Fig. 1:**
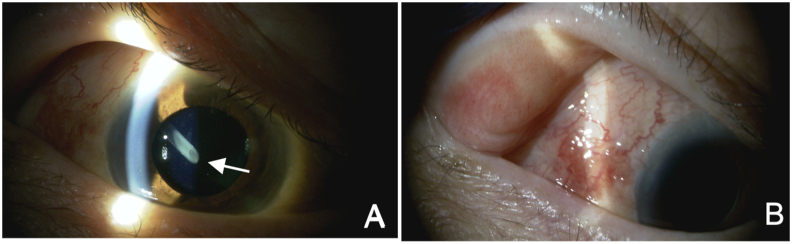
**Case 1, OD.** Slit lamp findings (A, B) 2 months after the AGV implantation. (A) AGV tube tip inserted via pars plana is seen behind the IOL (white arrow). (B) Superficial examination of superotemporal quadrant, where the plate of the AGV is implanted. No enlarged bleb is observed anterior to the front edge of AGV plate.

**Fig. 2:**
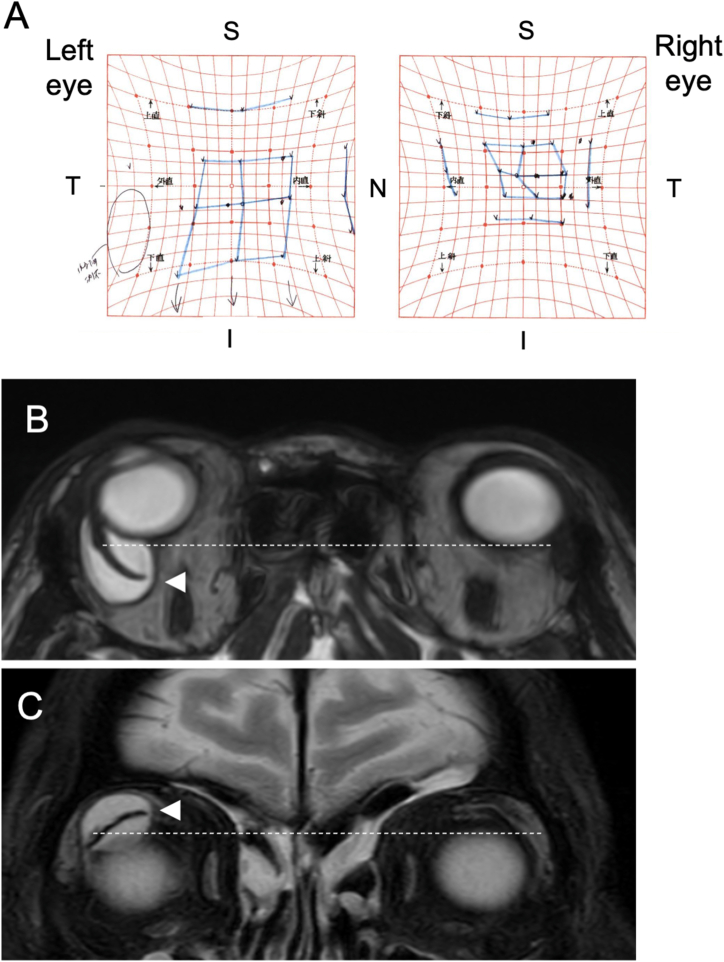
**Case 1.** Hess chart (A) and MRI of the orbit (B, C) findings 2 months after the AGV implantation surgery. (A) Hess chart evaluation shows the internal and inferior deviations OD in a primary eye position, restricted inferior an internal movements OD and the upgraded inferior and external compensated movements OS. (B) Axial view, T2-weighted orbital MRI, showing anterior displacement of eyeball due to a posteriorly enlarged giant bleb in OD. Aqueous humor appears as high intensity; AGV plate as low intensity (arrowhead), and the dotted line represents the posterior border of the eyeball OS. (C) Coronal view, T2-weighted orbital MRI illustrating inferior displacement of the eyeball due to a superiorly enlarged giant bleb in OD. Aqueous humor appears as high intensity; AGV plate as low intensity (arrowhead), and dotted line represents the superior border of the eyeball OS.

**Fig. 3:**
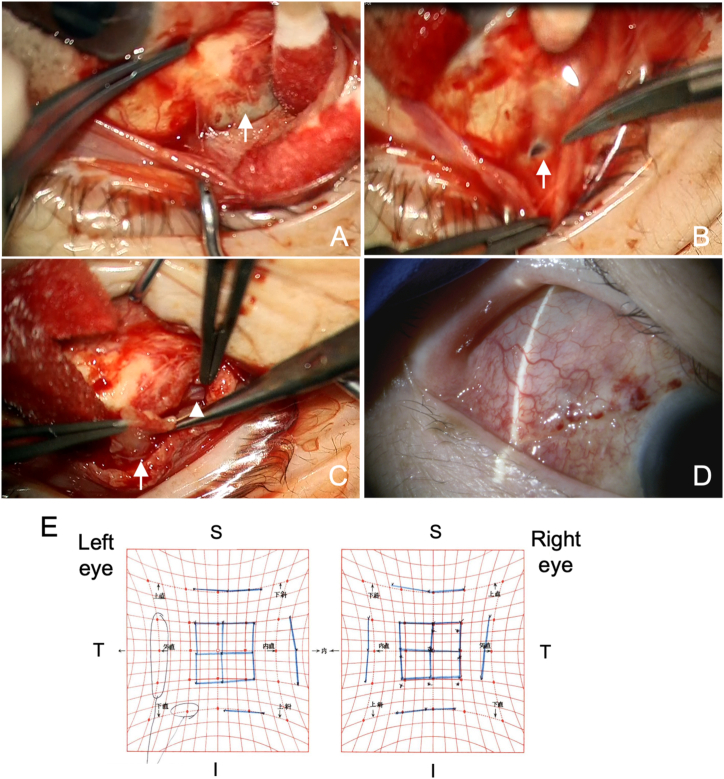
**Case 1.** Findings during surgery (A, B, C) and 3 weeks after surgery (D, E). (A) Debriding of the bleb wall is seen, with separation from conjunctival tissue (arrow) (B) An incision is made over the bleb wall to drain its content, with liquid beginning to flow out (arrow). (C) Excision of the anterior bleb wall (arrow), showing part of the AGV plate (arrowhead). (D) Slit lamp evaluation of the superotemporal quadrant of the ocular surface with the implanted plate shows normal bleb formation. (E) Hess chart evaluation demonstrates almost bilateral orthophoria.

A 70-year-old Japanese man with neovascular glaucoma due to diabetic retinopathy in his right eye (OD) underwent implantation of an Ahmed Glaucoma (AGV) (model FP-7, JFC Sales plan Co. Ltd., Tokyo, Japan) to reduce the intraocular pressure (IOP). Simultaneously, pars plana vitrectomy (PPV) and epiretinal membrane (ERM) peeling, along with removal of proliferative membrane were performed. Preoperatively, the IOP and best corrected visual acuity (BCVA) were 23 mmHg and 0.6, respectively, OD with the use of topical prostaglandin, beta-blocker and carbonic anhydrase inhibitor. The AGV plate was positioned in the superotemporal quadrant by placing the sutures at 8.5 mm from the corneal limbus, and the tube was inserted 3.5 mm away from the corneal limbus into the vitreous cavity, covered with an autologous scleral flap. The surgery concluded without complications. Preoperatively, 1.5% levofloxacin eye drops were used for 3 days, and postoperatively, 1.5% levofloxacin eye drops and 0.1% betamethasone eye drops were used for about 3 weeks. At the end of surgery, about 10 mg of triamcinolone was injected around the AGV plate. No antifibrotic agent was used during and after the surgery. Thirteen days post-surgery, IOP and BCVA were 9 mmHg and 0.8, respectively, OD. After two months, the IOP and BCVA were 24 mmHg and 0.8, respectively, OD, and the patient began experiencing double vision. Slit lamp examination displayed no abnormality beyond the typical findings after AGV implantation combined with PPV (Fig. 1A and B). The Hess chart exhibited esotropia and hypotropia OD upon comparison to the position of the central point in the left eye (OS), limited infraduction and adduction OD and compensatory enhanced infraduction and abduction OS (Fig. 2A). For further assessment, orbital magnetic resonance imaging (MRI) was conducted. T2-weighted orbital MRI,^5^ revealed a posterior cystic fluid accumulation surrounding the AGV in the superotemporal quadrant, leading to displacement of the right eyeball. Axial view (T2-weighted orbital MRI) anterior displacement of eyeball due to a giant bleb enlarged posteriorly was seen OD (Fig. 2B). Coronal view of the T2-weighted orbital MRI displayed inferior displacement of eyeball due to an enlarged superiorly extending bleb (Fig. 2C). Based on these findings, the patient underwent surgical intervention involving excision of the bleb wall (Video 1). No glaucoma medications were used preoperatively. The procedure began by debriding the bleb wall, separating it from the conjunctival tissue (Fig. 3A), followed by incision of the bleb wall to drain its contents (Fig. 3B). Subsequently, the anterior bleb wall was excised (Fig. 3C), and the bleb wall and conjunctiva were sutured separately. No antifibrotic agent was used during and after the revision surgery. The day after the procedure, the IOP was 8 mmHg OD. Topical application of levofloxacin 1.5% (Nipro, Osaka, Japan) and betamethasone 0.1% (Sanbetason, Santen Pharmaceutical, Osaka, Japan) four times daily for three weeks was prescribed. Aqueous suppression was not used after the surgery. By three weeks after surgery, the double vision had resolved (Fig. 3E). At the two-month follow-up, the IOP and BCVA were 17 mmHg and 0.7, respectively, OD. Slit lamp examination revealed a normal bleb (Fig. 3D).

### 2.2 Case 2

**Fig. 4:**
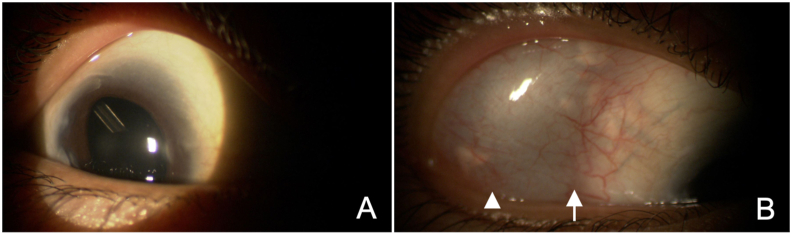
**Case 2, OD**. Findings in slit lamp 7 month after surgery (A, B) (A) AGV tube tip inserted via pars plana is seen behind the IOL (white arrow). (B) The bleb is clearly visible due to anterior enlargement during slit lamp examination. The anterior edge of the bleb located more anteriorly than the front edge of the AGV plate (arrowhead).

A 10-year-old Japanese female with bilateral aphakia (due to congenital bilateral cataracts), microcornea, nystagmus, and childhood glaucoma, receiving maximum therapy (prostaglandin plus timolol plus dorzolamide), presented with an IOP of 25 mmHg and BCVA of 0.5, OD. She underwent PPV in addition to AGV implantation, OD. The AGV plate was placed in the superotemporal quadrant by placing the sutures at 8.5 mm from the corneal limbus, and the tube was inserted into the vitreous cavity 3.5 mm away from the corneal limbus, covered with an autologous scleral flap. Preoperatively, 1.5% levofloxacin eye drops were used for 3 days, and postoperatively, 1.5% levofloxacin eye drops and 0.1% betamethasone eye drops were used for about 3 weeks. At the end of surgery, about 10 mg of triamcinolone was injected around the AGV plate. No antifibrotic agent was used during and after the surgery. Aqueous suppression was not used after the surgery. On the following day, the IOP and BCVA measured 5 mmHg and 0.4, respectively, OD. Seven months post-surgery, slit lamp evaluation indicated the well-positioned AGV tube inserted via the pars plana and positioned behind the intraocular lens (IOL) in the vitreous cavity (Fig. 4A). However, an anterior bleb enlargement was also observed, extending more anteriorly than the front edge of the AGV plate (Fig. 4B). At this point, the IOP and BCVA were 20 mmHg and 0.3, respectively, OD. This anterior enlargement of the bleb did not exert pressure on the eyeball and solely posed a cosmetic concern, with the patient experiencing no other symptoms.

## 3 Discussion

The occurrence of giant blebs as a secondary outcome of AGV implantation is a relatively uncommon complication, and it often goes unnoticed in large-scale clinical studies.^6^, ^7^, ^8^ Through our cases and the analysis of previously documented cases, we have identified two distinct types of giant blebs: anterior enlargement and posterior enlargement (Table 1). In the anterior enlargement type, the edge of the bleb extends more anteriorly than the front edge of the AGV plate (Fig. 4B). This anteriorly expanding bleb is easily observable during slit lamp examination and can lead to patient-reported concerns such as cosmetic issues, foreign body sensations, and pain, as exemplified in Case 2. Conversely, in the latter type, where the bleb enlarges posteriorly, there may not be any particular discernible findings during regular slit lamp evaluations (Fig. 1A and B). Patients with this type can report symptoms like dysesthesia, progressive proptosis, and diplopia, as demonstrated in Case 1. In these instances, T2-weighted orbital MRI becomes a valuable tool for gauging the extent of the bleb within the orbit (Fig. 2B and C).

**Table 1:** Giant bleb formation after AGV.

	ANTERIOR	POSTERIOR
Definition	Anterior enlargement of the bleb	Posterior enlargement of the bleb
Symptoms/Findings	･ Cosmetic problem	･ Dysesthesia
	･ Foreign body sensation/pain	･ Proptosis
	･ Dellen formation at the peripheral cornea/limbal conjunctiva	･ Limitation of eye movement
	･ Ptosis	･ Diplopia
Appearance of bleb by slit lamp	･ Anterior enlarged bleb is easily observed	･ No enlarged bleb is observed anterior to the front edge of AGV plate
	･ Edge of the bleb is located more anteriorly than the front edge of AGV plate	
Evaluation examinations	･ Slit lamp	･ Orbital MRI (T2)
	･ Orbital MRI (T2)	･ Hess Chart
Indication for surgical treatment	･ Symptoms/Findings are clinically remarkable	･ Symptoms/Findings are clinically remarkable
	･ Poor IOP control due to bleb encapsulation	･ Poor IOP control due to bleb encapsulation
Potential surgical treatment	･ Bleb wall recession technique	･ Anterior bleb wall excision technique

In a prior study, Jeon et al. documented a case of posttraumatic glaucoma treated with superotemporal AGV implantation. Two years after the procedure, the patient complained of pain and progressive proptosis. Orbital MRI revealed a substantial reservoir in the superotemporal region of the orbit near the lacrimal gland fossa, characterizing a case consistent with the posterior enlargement type of giant bleb.^2^ Similarly, in Case 1 of our report, the patient experienced double vision, a phenomenon previously associated with glaucoma drainage device (GDD) implantation.^7^^,^^9^ Larger bleb size has been reported to be associated with diplopia.^10^ Notably, in Case 1, the presence of a posteriorly enlarged bleb resulted in a mass effect on the ocular globe and the extraocular muscles, leading to diplopia and disruption of ocular motor function.

In the second case, even though an anteriorly enlarged bleb was observed during slit lamp examination, the patient remained asymptomatic and was under routine follow-up. Notably, Younger et al. reported a case involving a filtering bleb extending into the upper eyelid after Baerveldt implant surgery.^4^ Manabe et al. reported a scenario where an open-angle glaucoma patient underwent AGV implantation in the superotemporal quadrant.^3^ Around 4 weeks following the procedure, a substantial conjunctival cyst formed in the same quadrant.^3^ This case presented with symptoms of foreign body sensation, pain, and dellen formation at the limbus/conjunctiva adjacent to the anterior border of the large cyst.^3^ The symptoms resolved after surgical intervention.^3^ Consequently, these two previous cases can be categorized as the anterior enlargement type of giant bleb. Danesh-Meyer et al. documented a case of posttraumatic glaucoma, characterized by symptoms and findings such as progressive proptosis, limited elevation and abduction, exposure keratitis, and cosmetic concerns. Orbital MRI revealed a significant reservoir in the superotemporal quadrant.^1^ This case showcases features that might overlap with both anterior and posterior types. The symptoms and findings shown in Table 1 may also be overlapped by the two types of giant blebs.

The complexity and variability in the presentation of giant blebs underscore the importance of careful assessment and tailored management strategies for each individual case. In Case 2 and the previously reported cases,^1^^,^^4^ surgical intervention was not pursued. Hence, when patient symptoms and findings are mild and IOP is well managed, a conservative treatment approach becomes the primary consideration. However, when patient symptoms and findings are pronounced and/or IOP control is suboptimal, a surgical approach might be warranted, as evidenced in our Case 1 and a previously documented case.^3^ For instances like the previously reported case with anterior giant bleb enlargement,^3^ a surgical technique involving the posterior repositioning of the anterior bleb wall (known as the bleb wall recession technique) was employed. In this case, just a week after the surgical intervention, the bleb size diminished, ocular pain subsided, and effective IOP control was achieved.^3^ Conversely, in our Case 1 with posterior giant bleb enlargement, the excision of the anterior bleb wall (termed the anterior bleb wall excision technique) was employed due to the challenging accessibility of the posterior bleb edge for surgical manipulation. Identifying the direction of enlargement may be helpful in determining the appropriate treatment strategy. Anterior bleb wall excision technique may be effective for anterior giant blebs, but we have no experience with it. The variability in cases underscores the need for individualized management approaches.

To our knowledge, prior to this report, no classification system for giant bleb enlargement following GDD implantation had been established in the literature. Through the presentation of these two cases, we have now identified and classified two distinct types of giant bleb enlargement based on the direction of the expansion of the bleb. This classification provides valuable insights that enable treating physicians to differentiate and understand the underlying variations in these cases. Considering this classification and evaluating patient symptoms and findings, clinicians are advised to make informed decisions regarding the most suitable treatment approach. The understanding of these different types of giant bleb enlargement can guide tailored and effective management strategies for improved patient outcomes.

### 3.1 Patient consent

The patient provided written informed consent for publication of this case report and any accompanying pictures.

## Funding sources

No financial support was provided.

## Authorship

All authors attest that they meet the current ICMJE criteria for Authorship.

## Data availability statement

All data generated or analyzed during this study are included in this article. Further enquiries can be directed to the corresponding author.

## Artificial intelligence statement

The English grammar and wording were reviewed by Chat GPT 4.

## CRediT authorship contribution statement

**Ricardo Ugarte:** Writing – original draft, Investigation, Data curation, Conceptualization. **Kazunobu Sugihara:** Writing – review & editing, Investigation, Data curation. **Ichiya Sano:** Writing – review & editing, Investigation, Data curation. **Kana Murakami:** Writing – review & editing, Investigation, Data curation. **Mizuki Iida:** Writing – review & editing, Investigation, Data curation. **Ayaka Shimada:** Writing – review & editing, Investigation, Data curation. **Sho Ichioka:** Writing – review & editing, Investigation, Data curation. **Akiko Harano:** Writing – review & editing, Investigation, Data curation. **Masaki Tanito:** Writing – original draft, Investigation, Data curation, Conceptualization.

## Declaration of competing interest

Masaki Tanito received honorarium and research donations from JFC Sales plan Co. Ltd., Tokyo, Japan. Other authors have no conflicts of interest associated with this report.
